# Plant Responses to Pathogen Attack: Small RNAs in Focus

**DOI:** 10.3390/ijms19020515

**Published:** 2018-02-08

**Authors:** Waqar Islam, Ali Noman, Muhammad Qasim, Liande Wang

**Affiliations:** 1 College of Plant Protection, Fujian Agriculture and Forestry University, Fuzhou 350002, China; waqarislam@m.fafu.edu.cn (W.I.); qasim_gill54@yahoo.com (M.Q.); 2 Department of Botany, Government College University, Faisalabad 38040, Pakistan; alinoman@gcuf.edu.pk; 3 College of Crop Sciences, Fujian Agriculture and Forestry University, Fuzhou 350002, China

**Keywords:** non-coding RNA, plant diseases, genetic control, immunity, defense response

## Abstract

Small RNAs (sRNA) are a significant group of gene expression regulators for multiple biological processes in eukaryotes. In plants, many sRNA silencing pathways produce extensive array of sRNAs with specialized roles. The evidence on record advocates for the functions of sRNAs during plant microbe interactions. Host sRNAs are reckoned as mandatory elements of plant defense. sRNAs involved in plant defense processes via different pathways include both short interfering RNA (siRNA) and microRNA (miRNA) that actively regulate immunity in response to pathogenic attack via tackling pathogen-associated molecular patterns (PAMPs) and other effectors. In response to pathogen attack, plants protect themselves with the help of sRNA-dependent immune systems. That sRNA-mediated plant defense responses play a role during infections is an established fact. However, the regulations of several sRNAs still need extensive research. In this review, we discussed the topical advancements and findings relevant to pathogen attack and plant defense mediated by sRNAs. We attempted to point out diverse sRNAs as key defenders in plant systems. It is hoped that sRNAs would be exploited as a mainstream player to achieve food security by tackling different plant diseases.

## 1. Introduction

With an increasing global population and shrinking agricultural lands, it is mandatory to increase world food production [1]. Biotic threats to food security include pathogens (bacteria, fungi, nematodes, oomycetes and viruses) as well as insect pests. Cumulatively, these are responsible for an approximately 30% loss in global crop production at pre- and post-harvest levels [2]. All plant pathogens continuously challenge the host immune system [3], so, to counteract multifarious infection types, plants have evolved an array of defense responses by activating or suppressing a number of genes [4,5,6]. In succeeding events of pathogen attack, plants recognize a cascade of PAMPs or DAMPs (host danger-associated molecular patterns) [3,7,8,9]. Cell-surface-based PRRs (pattern-recognition receptors), e.g., FLS2, EF-Tu, LYK5, and CERK1, perceive pathogens, i.e., bacteria- and fungi-triggered PAMPs or DAMPs [10,11,12,13]. PAMP as well as DAMP triggers PTI (PAMP-triggered immunity) involving the induction of PR (pathogenesis-related) gene expression, callose deposition, the production of ROS (reactive oxygen species), and SA (salicylic acid) accumulation [14,15]. However, during the course of evolution, many pathogens have evolved effector proteins used for suppressing PTI and culminating in effector-triggered susceptibility (ETS) [16,17,18]. In response, the plant secondary immune response, commonly called as effector-triggered immunity (ETI), is used as a defense. Moreover, novel resistance (R) proteins are utilized by the plants to recognize the effectors and trigger ETI responses in this endless arms race [19]. R proteins usually result in a more vigorous and precise reaction such as a hypersensitive response (HR) [20]. An HR reconciles infection by causing cell death at the infected sites by restricting pathogen growth. Against diversified effectors as a result of defense and counter-defense between hosts and pathogens, plants have evolved new R proteins to distinguish and tackle new effectors [21,22,23].

The non-coding RNA molecules that are 20–30 nucleotides (nt) long regulating eukaryotic gene expression through RNA silencing are known as sRNAs [24,25,26]. In plants, sRNAs are classified into two major categories termed as miRNA and siRNA, respectively. miRNAs are 21–24 nt long and generated from RNAs with imperfectly base-paired hairpin structures [27,28,29,30,31,32,33,34]. siRNAs are obtained from long double-stranded RNAs (dsRNAs) and may need RNA-dependent RNA polymerases (RDRs) [35,36]. Several sub-classes of siRNA have been reported in plants, inclusive of ta-siRNAs (*trans*-acting siRNAs), hc-siRNAs (heterochromatic siRNAs), nat-siRNAs (natural antisense transcript-derived siRNAs), and lsiRNAs (long siRNAs) [37]. Post-transcriptional gene silencing (PTGS) or transcriptional gene silencing (TGS) is mainly responsible for sRNA-mediated gene regulation in hosts or pathogens [38]. miRNAs and siRNAs can induce PTGS by mRNA (messenger RNA) cleavage/degradation or translational repression through RISC (RNA-induced silencing complex) [5,39]. Contrarily, TGS, ending at either DNA methylation, histone modification, or chromatin modification, is generally mediated by siRNAs and some specific miRNAs as well [40,41]. The biogenesis of different sRNAs is complex and species-specific, and in spite of some common steps, many steps are exclusive for certain sRNAs. Regarding the biogenesis of various sRNAs, much data are available [39,42,43,44,45,46,47]. Therefore, due to the immense implication of sRNAs in plant immunity, we have summed up the targeted functions of sRNAs as front line players in mediating plant growth under pathogenic pressure. We mention recent advancements in sRNA research, particularly explaining their role as a dynamic network in mediating plant immunity against pathogens.

## 2. Plant sRNAs against Pathogen Attack

**Figure 1:**
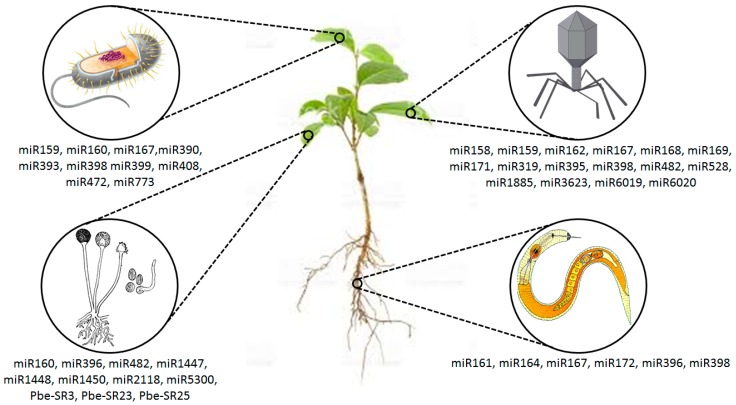
The role of small RNAs (sRNAs) against infection of various pathogens (general illustration).

Different plant diseases due to phyto-pathogens cause substantial damages to crop production and ultimately result in heavy economic losses [6]. These pathogens include bacteria, fungi, mycoplasma, nematodes, viruses, viroids, and parasites. Various groups of plant sRNAs play vital roles in plant defense against pathogens. sRNAs involved in plant defense processes via different pathways include both siRNA and miRNA that actively regulate immunity in response to pathogenic attack via tackling PAMPs and other effectors [48,49,50,51,52] (Figure 1). Against pathogen attack, up-/down-regulation of sRNAs lead to the suppression of target site expression [37]. However, regulations of several sRNAs still need extensive research. Multiple proteins take part in sRNA pathways for initiating successful defense response to the pathogens through sRNAs biogenesis and functions. These proteins include (a) endoribonuclease DICER or DICER-like (DCL) involved in the production of sRNAs, (b) argonautes (AGOs) performing sRNA-directed gene suppression, and (c) RNA-dependent RNA polymerase (RDRs) taking part in the genesis of dsRNA precursors [53]. The genome of *Arabidopsis thaliana* encodes four proteins in which DCL1 is regarded as the vital one in connection with miRNA genesis. Several miRNAs are related to ETI and PTI against fungal and bacterial attacks. *A. thaliana* mutants, i.e., *dcl1-9* [54] and *dcl1-7* [55] were found to be susceptible to pathogen infections. This illustrates the regulation of immune responses through miRNAs. Other proteins like DCL4 are liable to siRNA generation and is important against pathogen attack, e.g., bacteria, fungi, and viruses [56,57]. AGOs also regulate the immune process. For example, bacterial infection in *A. thaliana* results in the generation of AGO2, so its mutant *ago2-1* in tomato embedding miRNA393 is considerably susceptible towards several prominent strains of *Pseudomonas syringae* [58]. It is interesting to note that miRNA393 works through AGO1 for the suppression of auxin receptors for the activation of anti-bacterial immunity [59]. AGO1 mutants (*ago1-25* and *ago1-27*) actively acquired immunity against bacteria. Of note, these mutants were also found to be resistant to fungal infection [60,61]. The *A. thaliana* genome possesses six RDRs. RDR6 produces secondary siRNA, i.e., ta-siRNA. The *rdr6* mutant is highly susceptible to fungus [60] but is resistant to *Pseudomonas* strains [62]. Furthermore, mutations due to the interface of RDR6 and antiviral proteins (SGS3) also enhance the susceptibility towards *Verticilium* infections [60]. Thus, it can be inferred that sRNA pathways are more important in the functioning of anti-fungal defenses in plants.

DNA methylation and histone modification are directed through hc-siRNA and induces transposons silencing, palindromic repeats, and genes at the transcriptional stage. This RNA-directed DNA methylation (RdDM) pathway is also involved in immunity regulation [63,64,65]. Mutants materialized through RdDM show phenotypic disease modifications against bacterial and fungal infections. This is supported by the fact that a triple mutant of the non-cytosine (CG loci) methyltransferases (*drm1-2/drm2-2/cmt3-11*) with POL-IV subunit presented high resistance to *P. syringae* virulence [66]. Likewise, low expressions of different RdDM pathway proteins as a result of *P. syringae* virulence have been observed. PAMP-activated *flg22* supports the view that antibacterial defense gene expression is transcriptionally regulated by RdDM [67]. Contrarily, ROS1 encoding for 5-methylcestocine DNA glycosylase that initiates active DNA demethylation is repressed upon flg22 treatment, and its mutants are greatly susceptible to *P. syringae* infections [67]. This favors the view that defense tactics against pathogen attacks cause active DNA demethylation which is a part of the regulatory circuit for gene activation in response to pathogen attacks. *Arabidopsis* RdDM mutants (*nrpe1 & ago4*) and *rdd*, i.e., triple DNA demethylase mutant (*ros1/dml2; DEMETER-LIKE 2/dml3; DEMETER-LIKE 3*) revealed susceptibility against *Fusarium oxysporum* attack [65]. Obviously, DNA demethylation or RdDM re-arranges the transcriptional status of various immunity genes. Enhanced susceptibility against necrotrophic fungal pathogens (*Botrytis cinerea* and *Plectospherella cucumerina*) was observed in the *ago4*, *drd1*, *rdr2*, *drm1 drm2*, and *nrpd2* mutants, which is contrary to the increased resistance against bacterial pathogens [68]. For this reason, sRNA and their associated pathways are critical factors in regulating the host immunity towards pathogen attack.

## 3. Plant Small RNAs against Viruses and Viroid Infections

**Figure 2:**
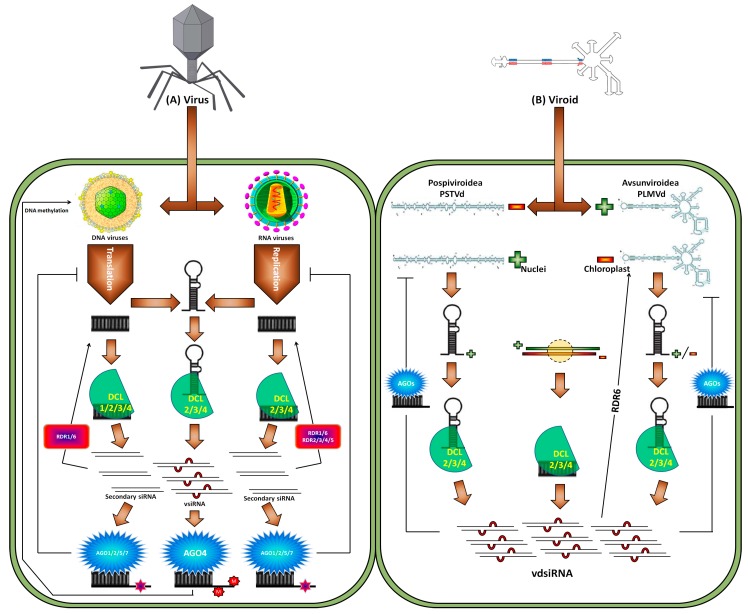
(**A**) The role of sRNAs against viruses and viroid infections in plants. (**A**) Numerous virus-derived small interfering RNAs (vsiRNAs) are generated in plants that directly target the viral genomes to initiate defense against virus infections. The generation of vsiRNA regarding DNA viruses and RNA viruses are explained here. Regarding DNA viruses, the vsiRNAs are processed via the structural region of the transcripts along with the overlapping regions of the bi-direction transcription. On the contrary, upon the infection of RNA viruses, the structure region of viruses can be processed through DCL proteins. However, in both cases, secondary vsiRNAs are coordinated by RDR1 and RDR6. After production, these vsiRNAs are loaded into different argonautes (AGOs), which further play a role in virus genome silencing. vsiRNAs silence the genomic RNA upon RNA virus infection. However, upon the infection of DNA viruses, DNA methylation is initiated through these vsiRNAs. (**B**) *Potato spindle tuber viroids* (PSTVds) are found in nucleolus and their viroid-related siRNAs (vdsiRNAs) show predominant mapping to the positive strand of the left and right terminal regions. It is thought that the generation of these vdsiRNAs is linked to the hairpin loopy structure of positive strand of PSTV transcripts. The secondary structure of PSTVd transcripts are targeted by DCL protein and sliced into vdsiRNA. vdsiRNA can also be generated by the accidental association of positive and negative strand replication, further regulated by DCL proteins. Oppositely, PLMVds replicate inside the chloroplast and have the ability to generate vdsiRNAs from both positive and negative strands. The stem-loopy or hairpin-like structure of PLMVd is processed through various DCL proteins to produce vdsiRNAs. It is also illustrated that amplification of vdsiRNAs through RDRs is also possible, and the generation, the vdsiRNA can be loaded into plant AGO proteins leading towards the targeting of viroid RNAs.

Plants are affected by viruses and viroids due to their replicating genomes in the host cells [69,70,71]. PTGS was primarily recognized in transgenes with *Potato virus X* (PVX) infection. During investigation, sRNAs complementary to the sense transcript of the transgene and the positive strand of PVX were revealed, which indicates sRNA participation in viral defense and PTGS transgene silencing [72,73]. Studies have demonstrated virus and viroid replication along with RNA genomes and transcripts folding to generate dsRNAs that might play a role in RNA silencing machinery [74] (Figure 2).

Viruses possess ssRNA, dsRNA, ssDNA, or dsDNA genomes [26,75,76]. During the replication of an ssRNA viral genome, a complementary strand of RNA is synthesized, which forms a long dsRNA with the original viral genome. The dsRNA replication intermediate ssRNA types and the dsRNA genomes of dsRNA viruses can be targeted by a host RNA silencing apparatus [53,77]. Approximately equivalent quantities of positive- and negative-stranded virus-derived small interfering RNAs (vsiRNAs) without positional bias were derived from different families such as *Cucumber yellows closterovirus* (CuYV), *Turnip mosaic potyvirus* (TuMV), *Cucumber mosaic virus* (CMV), *Watermelon mosaic virus* (WMV), PVX, and *Tomato yellow leaf curl virus* (TYLCV) [78,79,80,81,82]. Interestingly, positive and negative strands of *Rice stripe virus* (RSV) generated an equal amount of vsiRNAs [83]. On the other hand, over 80% of vsiRNAs produced from the positive strand have been obtained from *Cymbidium ringspot virus* (CymRSV) [84,85]. Similar phenomena are evident in plants infected with other ssRNA viruses, i.e., TCV, TMV, TRV, and PMMoV (*Pepper mild mottle virus*). In these examples, some positive strand vsiRNAs accounted up to 97% of total vsiRNAs [79,81,86]. There are no dsRNA intermediate replicative forms for ssDNA and dsDNA viruses. Some of the vsiRNAs from DNA viruses show that vsiRNAs might be processed from the structured part of the viral RNA transcripts. Sixty-two percent of the vsiRNAs resemble the CaMV (*Cauliflower mosaic virus*) transcript polarity, a common source of constitutive 35S promoter. Nearly 82% vsiRNAs are synthesized from the leader region, with no strand bias [37,87]. A geminivirus, namely *Tomato yellow leaf curl China virus* (TYLCCNV), possess an ssDNA genome. Although the vsiRNAs generated from TYLCCNV present a site bias, they map approximately equal to the negative and positive genomes [83,88]. Consequently, dsRNA replicative forms and the viral genomes secondary structure can be processed by host RNA silencing apparatus. However, the finalized opinion on viral pathogenicity with respect to these findings is still unknown.

Viroids, the smallest pathogens replicating inside the nucleus or chloroplast, are comprised of naked, single-stranded, and circular RNAs that are 250–400-nt in size [74]. Two decades ago, full methylation of the *Potato spindle tuber viroid* (PSTVd) cDNA sequence was observed in PSTVd-infected tissues [89,90]. The viroid-induced RNA silencing and RdDM are responsible for this methylation. Later, it was confirmed that viroids are the activator and target of RNA silencing in infected tomato and tobacco plants [91,92]. Viroid-related siRNAs (vdsiRNAs) of PSTVd are produced from polarities in the right and left domains. With the help of deep sequencing profiling of PSTVd vdsiRNAs, it was revealed that PSTVd vdsiRNAs chiefly map to the positive strand of the right and left terminal regions. This observation indicates that these sRNAs are produced from the secondary structure of positively stranded RNAs. A number of vdsiRNAs are also produced by the negative strand of the central part presenting their probable processing from the secondary structure of the negative-strand viroid genomic RNA [93]. *Citrus exocortis viroid* (CEVd) after replication mainly generates 5′-phosphorylated and 3′-methylated vdsiRNAs with positive polarity. Most CEVd vdsiRNAs are localized within the right-end domain. This proposes that structured RNA is the vital substrate of DCL enzymes [94,95]. Some viroids such as *Chrysanthemum chlorotic mottle viroid* (CChMVd), *Peach latent mosaic viroid* (PLMVd), and *Avocado sunblotch viroid* (ASBVd) replicate in the chloroplast. Both PLMVd and CChMVd produce vdsiRNAs from negative and positive polarities [96,97]. ASBVd also produces vdsiRNAs in leaves exhibiting infection symptoms [98]. Therefore, both viroid families, i.e., *Avsunviroidae* and *Pospiviroidae*, possess a capacity to generate vdsiRNAs in different plants [99,100]. Generation of vdsiRNAs from both the positive and the negative strands of the viroid genome highlight the predominant processing of vdsiRNAs from the viroid genomic RNAs secondary structure. Nevertheless, it is pertinent to indicate that the biasness in discoveries of vdsiRNAs depends upon the methods applied for sRNA cloning.

The plant DCL proteins process inducers of dsRNA for production of sRNAs. The model plant *Arabidopsis* possesses four DCL proteins that are used to generate diverse sRNAs [43]. DCL1 cleaves hairpin primary micro RNAs (pri-miRNAs) into a smaller stem-loop structure called precursor microRNAs (pre-miRNAs), which are subsequently processed again by DCL1 to produce mature miRNA duplexes consisting of the active miRNA strand and its complementary strand miRNA [101]. DCL2–4 produces siRNAs with specific sizes (22, 24, and 21 nt) from long perfect dsRNAs [102]. For instance, DCL2, -3, and -4, respectively, produce nat-siRNAs, hc-siRNAs, and ta-siRNAs, but DCL4, -2, and -3, redundantly produce viral siRNAs to defend against virus infection [103,104,105,106]. Predominant dsRNA inducers for RNA viruses and viroids include the perfectly paired dsRNA intermediate replication form and the hairpin structure of the single genomic RNA. Indeed, DCL2, -3, and -4 are accountable for processing ssRNA viruses, e.g., CMV, TuMV, and TCV, into 21, 22, and 24 nt vsiRNAs, respectively [107,108,109,110,111]. The new leaves in PSTVd-infected plants simply accrue small (21–22 nt) vdsiRNAs, in contrast to the older leaves having shorter as well as longer (24 nt) vdsiRNAs [112,113]. Analogous vdsiRNA buildup patterns have been observed in plants attacked by *Hop stunt viroid* (HSVd) and *Hop latent viroid* (HLVd). However, very few reports are available regarding vdsiRNAs biogenesis. The accumulation of 21, 22, and 24 nt vsiRNAs has also been recorded in plants infected with *Beet curly top virus* (BCTV), *Cabbage Leaf Curl Virus* (CalCuV), and *Pepper golden mosaic virus* (PepGMV). Notably, all of these are ssDNA viruses except CaMV, which is a dsDNA virus [87,114,115]. From DNA viruses, the predominant production of 24 nt vsiRNAs is on record. As stated earlier, DCL proteins like DCL2, DCL3, and DCL4 are crucial for the accumulation of different CalCuV vsiRNAs. Plants infected with BCTV, DCL3, and DCL4 were found to be responsible for amassing 21 and 24 nt vsiRNAs [115]. In spite of the mandatory role of the hairpin structure of viral or viroid genomes for the production of vsiRNA and vdsiRNAs, DCL1-dominant hairpin processing is not involved in vsiRNA and vdsiRNAs accumulation or anti-RNA-viral resistance. However, DCL1, not DCL4, is needed for the increase in 21 nt vsiRNAs from CaMV [87].

Following the early processing of dsRNA inducers, both the antiviral and anti-viroid signals are amplified by host RDRs. In *A. thaliana*, out of six RDRs, the functions of RDR1, RDR2, and RDR6 are well studied. SA and TMV infection induce RDR1 in tobacco and *Arabidopsis.* A mutation in RDR1 allows well-organized development of ssRNA viruses [116,117]. Moreover, the *AtRDR6* mutant appeared highly susceptible to the attack of ssRNA and ssDNA viruses [118]. Decreased expression of rice RDR6 was observed after infection with RSV and RDV (a negative ssRNA virus and a dsRNA virus, respectively). Additionally, *OsRDR6* down-regulation by antisense transformation caused a high susceptibility to RDV [119,120,121]. It has also been noted that RDR1 and RDR6 are imperative for secondary CMV vsiRNA genesis in *A. thaliana.* Further explaining CMV, Quintero et al. [122] employed the bioinformatic approach for the identification of ta-siRNAs and *cis*-nat-siRNAs in cassava plants from two sRNA libraries constructed by healthy cassava plants and *Xanthomonas axonopodis* pv. *manihotis* (*Xam*) inoculated plants. They identified 54 possible ta-siRNA loci in cassava. Out of these, 15 loci were induced, while the other 39 were repressed in response to *Xam* infection. Additionally, 15 possible *cis*-natural antisense transcript (*cis*-NAT) loci, involved in the production of siRNAs, were also identified from overlapping antisense regions in the genome. All of these were differentially expressing upon *Xam* infection [122]. RDR1 is needed for vsiRNAs production from the 5′-terminal end of the viral genome, while RDR6 is involved in the vsiRNAs production from the 3′-terminal ends [80]. However, *Nt*RDR1 expression in *Nicotiana benthamiana* plants, which do not encode RDR1, exhibited the suppression of RNA silencing mediated by RDR6 and enhanced infection in transgenic plants [123,124]. Moreover, a high accumulation of HSVd and PSTVd genomic RNAs in *rdr6*-silenced plants demonstrates the contribution that RDRs make in anti-viroid resistance. Systematic analysis by means of vsiRNA and vdsiRNA profiling in infected plants revealed that sRNAs were processed from pathogen genomic RNAs and declined in *rdr* knock-out mutants and silenced plants [94,119,123,125]. The lessened vsiRNA and vdsiRNA accumulation along with increased susceptibility of *rdr* mutant plants clearly advocates the anti-viral/viroid function of RDRs. Even though RDR2 is liable for accumulation of 24 nt hc-siRNA, *RDR2* mutation exerts little or no effect on vsiRNA accumulation of DNA viruses, e.g., CalCuV and CaMV [87]. Tomato *Ty-1* and *Ty-3*, TYLCV resistance genes, encodes RDRs with sequence similarity to *A. thaliana* RDR3, -4, and -5. The susceptible *Lycopersicum* lines without these loci witness lower levels of TYLCV vsiRNAs and accrue high viral titers [126,127,128]. Still in *Arabidopsis*, the complete antiviral attributes of RDR3, RDR4, and RDR5 have not yet been unveiled. Therefore, there is a dire need to study the function of RDR2 and other RDRs in host-virus/viroid interactions.

The replication and movement of viruses as well as viroids is restricted after vsiRNAs and vdsiRNAs are loaded into AGO proteins. AGO1, AGO2, AGO3, AGO5, AGO7, and AGO10 unite with vsiRNAs or participate in anti-viral RNA silencing pathways [37]. Resurgence from attack with a DNA virus entails the purpose of host AGO4 [129]. As a result, the 24 nt vsiRNAs of DNA viruses may bracket together with AGO4 for methylation of the viral genome. A mutant defective in double-stranded RNA-binding protein (DRB3) interacts with DCL3 and AGO4, shows lower methylation of the viral DNA genome along with greater hyper susceptibility to geminiviruses. It further shows the role that the DCL3-AGO4 RdDM pathway plays in modulating resistance against DNA viruses [130]. Additionally, a novel AGO, i.e., AGO18, is tempted by RSV and maintains rice antiviral resistance [131]. With reference to the vdsiRNAs, 21 and 22 nt vdsiRNAs predominately load into AGO1, AGO2, and AGO3 [37,132]. On the other hand, AGO4, AGO5, and AGO9 become the target of 24 nt vdsiRNAs for loading. AGO6, AGO7, and AGO10 do not bind vdsiRNAs [37]. However, the anti-viroid job of these AGOs needs further investigation.

Contrary to the DCL-based vdsiRNAs processing and loading into plant AGOs, regulation of viroid genomes is still unclear. Transgenic dsRNAs or co-inoculated dsRNAs can be used to silence PSTVd, CEVd, and CChMVd in plants. This silencing is sequence-specific, temperature-dependent, and dose-dependent [112,133,134]. Several classes of miRNAs have been discovered that are generated by plants in response to virus and viroid attacks. These play a key role in defense against various viral diseases (Table 1). However, it is evident that viroids may have evolved a mechanism against sRNA silencing. Based on PSTVd and HSVd studies, the viroid circular genome is resistant to RNA silencing [135].

## 4. Plant Small RNAs against Fungal Infections

With high throughput technology and advancements in bioinformatics, several sRNAs have been identified for their decisive roles in disease development in the case of fungal invasion. sRNAs interplay with multiple classes of disease resistance genes (Table 2) [163,164]. The host sRNAs and their respective targets, either by up- or down-regulation, are involved in disease severity after fungal attack [49,165,166]. Conversely, if plants carry the R-gene product, the resistance is administered by the R-Avr interface by holding onto the intrusion of sRNAs [167,168]. Moreover, plant sRNAs regulate homeostasis of plant growth regulators by managing the target transcripts expression [169,170]. miR408 is a negative regulator of plantacyanins and laccase [171,172]. Even though the exact role of plantacyanins in plants is mysterious; however, they are supposed to be involved in stress responses cell-to-cell signaling and lignin production [173,174]. Similarly, laccases regulate varied tasks in plants, e.g., lignin synthesis, wound healing, iron acquirement, stress response, and cell wall structure maintenance and veracity [175,176]. Therefore, differential regulation of miR408 in wheat cultivars susceptible and resistant to *Puccinia graminis* f. sp. *tritici* 2 days after inoculation (DAI) and 10 DAI [177] might result in plantacyanins and laccase-arbitrated perturbations in lignin biosynthesis and HR response. Similarly, miR2118 targets TIR-NBS-LRR *Verticillium-dahliae*-infected *Gossypium hirsutam* [163,178]. In the same way, pbe-SR23 and pbe-SR3 target TIR-LRR in *Populous* after *Dothiorella gregaria* attack [164,179]. Osa-miR7695 overexpression resulted in resistance to blast fungus. Os-miR7695-mediated negative regulation of natural resistance-associated macrophage protein 6 (*OsNramp6*) illustrated a novel regulatory network, integrating miRNA function and mRNA processing in plant immunity [180]. 

Yin and Li [163] conducted global identification studies of miRNAs and other sRNAs from *Gossypium barbadense* (*Verticillium*-tolerant cultivar) and (*Gossypium hirsutum* cv. Yi-11 (*Verticillium*-sensitive cultivar). Among 215 miRNAs families, over 65 miRNAs presented modified expression after *Verticillium* infection in both cotton species. Particularly, three *Populous*-specific miRNAs (Ptc-miR482, Ptc-miR482-1444, and Ptc-miR482-1448) target the cleavage of PPO (poly phenol oxidase) and R-protein genes regulating plant biotic and abiotic stress tolerance [181]. miR482 and miR1448 down regulation in *Verticillium*-infected cotton plants displayed amplified PPO and disease resistance proteins. TIR-NBS-LRR, a resistance protein for regulating pathogenesis, is reportedly a target of miR2118 [178,182]. The down-regulation of miR2118 shows a higher accumulation of corresponding target proteins, which could be a reason for the high defense response in cotton roots [163]. Chen and coworkers defined 74 conserved miRNA of 37 miRNA families and 27 novel miRNAs in *Populus beijingensis* infected with *D. gregaria* [164,183]. Among conserved ones, miR156, miR164, miR159, miR168, miR169, miR172, miR393, miR398, miR396, and miR1447 were up-regulated. Deep sequencing data indicated the 1.2-fold greater expression of conserved miRNA in infected plants compared to uninfected plants. This suggests that conserved miRNAs play important roles in defense. Some other miRNAs and siRNA have also been found to be up-regulated after infection with their putative target proteins participating in resistance to disease (Table 2). For example, miRNAs 1447, 1448, and 472 targeted disease resistance proteins and differentially regulated during *D. gregaria* infection [181,184]. In galled loblolly pine stems, miR156 expression was considerably suppressed under the attack of *Cronartium quercuum* f. sp. *fusiforme* [185]. Zhao et al. [165] identified pathogen responsive miRNAs in *Populus trichocarpa* infected with *Botryosphaeria dothidea*. They found that 41 fungi responsive up-regulated miRNA. Target prediction of these miRNAs indicated their participation in regulating cellular processes, including defense proteins, signaling surges, and metabolic pathways. The fungus responsive miRNAs target many genes at the same time, and each target gene is involved in several biochemical and physiological processes. As a result, the regulation and crosstalk of gene expressions during disease development actively helps to understand disease pathogenesis. 

Recently, sRNAs, playing an important role in enhancing immunity against the rice blast disease caused by *Magnaporthe oryzae*, were uncovered through deep sequencing of sRNA libraries from susceptible and resistant lines in normal conditions [186]. The experimentation revealed that a group of known rice miRNAs were differentially expressing upon *M. oryzae* infection. Furthermore, the real-time reverse transcription-polymerase chain reaction assay showed that the expression of some target genes was negatively correlated with the expression of miRNAs. The results showed that the transgenic rice plants overexpressing miR160a and miR398b embedded an enhanced resistance to *M. oryzae*, as the plants demonstrated decreased fungal growth along with increased hydrogen peroxide accumulation at the infection site and up-regulated expression of defense-related genes [186]. 

The unique expression pattern of miRNAs in the same family corresponds with their diverse functions in dissimilar species under different types of pathogen. Hence, to develop a comprehensive understanding of the regulatory functions of miRNAs and their target genes during pathogen attack, intense experimental validation of miRNAs are mandatory. For example, the miR1138 accumulation in infected wheat plants suggests that fungus perturbs the cell function homeostasis by modulating host protein biosynthetic machinery. In the presence of the *Sr24* gene, the expression of miRNAs decreased significantly depicting the R-gene mediated defense response and thus disease resistance [177,179]. Up or down-regulation of miRNA in the genotype having R-gene suggests interaction of miRNA with R-gene in disease signaling. In powdery mildew disease, the down-regulation of miR156, miR159, miR164, and miR168 suggests the up-regulation of corresponding target genes involved in signal transduction, stress response, root development, and oxidative stress response (Table 3).

## 5. Plant Small RNAs against Bacterial Infections

Plant sRNAs directly take part in responses to bacterial diseases (Table 4). The first identified sRNAs involved in plant immunity against bacterial infections were miRNAs. Bacterially infected plants exhibited several changes in miRNA accumulation and functioning, especially in auxin-signaling-associated miRNAs [54,193]. PAMP flagellin perception in *A. thaliana* restricted *P. syringae* invasion. However, no mechanism involved in triggering resistance is on record. Navarro et al. [194] observed the targets of miR393 with the help of gene expression profiles analysis in seedlings challenged with flg22. *TIR1*, *AFB2*, *AFB3* (the three auxin receptor transcripts) accumulation was suppressed upon flg22 treatment. However, *AFB1* was not perturbed, perhaps due to a somewhat dissimilar miR393-binding site. When the miR393-resistant auxin receptor *AFB1*-Myc was overexpressed in a *tir1-1* background, it enhanced disease susceptibility in *Arabidopsis*. The results suggested that miR393 was involved in controlling defense responses against *P. syringae*. Advanced experiments substantiated this role for miR393a. Overexpression of miR393a augmented plant resistance and trimmed down virulent *P. syringae* pv. *tomato* (Pst) DC3000 growth [194]. Captivatingly, both virulent and avirulent *P. syringae* strains harboring the type III effector protein, i.e., *avrRpt*2, demonstrated similar growth under the *AFB1*-Myc-overexpressing *Arabidopsis*. Accumulation of miR393 has also been reportedly induced in *Agrobacterium tumefaciens* infiltration. Fascinatingly, the flg22 of *A. tumefaciens,* completely inactive to the receptor kinase *FLS2* as a ligand, maintained miR393 expression levels without significant alterations [4,195,196]. Collectively, these results imply that miR393a is strongly involved in ETI and, most importantly, that repression of auxin signaling constitutes a plants defense response to bacterial infection.

High-throughput sequencing analyses support the up-regulated miR393 during plant–bacteria interactions [199,202,203,204]. Other than miR393, induction of sRNAs such as miR160 and miR167 has been observed after the inoculation of non-pathogenic Pst DC3000 *hrcC*^−^ and flg22. Surprisingly, treatment with flg22 did not presented a major drop of miR167 targets i.e., *ARF8* and *ARF6*, in contrast to the down-regulation of miR160 targets e.g., *ARF10*, *ARF16*, and *ARF17*. miR160a-overexpressing plants increased callose deposition after treatment with flg22 and *hrcC*^−^ mutant bacteria. Nevertheless, in plants overexpressing miR160, resistance to Pst DC3000 bacteria proliferation remained unaffected [61,198,205].

The accumulation of hormone-associated miRNAs contributes to plant defense, and the down-regulation of certain miRNAs influences plant immunity [48,61,198]. For instance, miR398, which is responsible for targeting copper superoxide dismutases (*CSD1*,*CSD2*) and a cytochrome c oxidase subunit V (*COX5*), is lessened in plants facing avirulent strains like *Pst* DC3000 *avrRpm1* and *Pst* DC3000 *avrRpt2* [182]. miR398 accumulation is also altered by abiotic as well as biotic stresses e.g., salinity, increased light, metals, ozone stress, and flg22 treatment [182,198,206]. During biotic and abiotic stresses, plants experience an accumulation of ROS in the infection site. Superoxide dismutases (SODs) convert superoxide into oxygen and hydrogen peroxide and therefore regulate ROS [1,207]. Expression of miR398 is reduced in oxidative stress, promoting accumulation of *CSD1* and *CSD2* [30,206]. In agreement with expression analyses under different stresses, overexpressing miR398 curtailed callose deposition after infection, i.e., flg22, Pst DC3000 *hrC*^−^. Moreover, these transgenic plants appeared more sensitive to virulent and avirulent strains of *P. syringae* after *CSD1*, *CSD2*, and *COX5* gene silencing [198]. Such observations confirmed a connection between the miR398 family and the miRNA-mediated plant defense responses.

miR773 takes part in PTI [198,208]. mRNA coding *DMT2* (DNA methyltransferase 2) is targeted by miR773. Crane and Gelvin [209] observed less tumor formation after silencing the *DMT2* gene during infection due to *Agrobacterium*. Deep sequencing analysis highlighted hampered miR773 accumulation after flg22 treatment. Accordingly, *DMT2* accumulation was induced against flg22 treatment. miR773-overexpressing transgenic plants had low methyltransferase 2 *(MET2)* mRNA levels, curtailed callose deposition, and high susceptibility to bacterial infections. Reduction of miR398 and miR773 under biotic stress points out a negative regulation of PTI [61,198,208].

In sRNA-based plant immune response, the starred strand of miR393* also functions in plant defense [151,210]. In *A. thaliana*, AGO2 had been induced after *P. syringae* attack. After *Pst avrRpt2* treatment, star strand e.g., miR393* was noticeably loaded into AGO2. Among predicted targets for miR393, a validated target that functions in vesicle transport is Golgi-localized SNARE protein, i.e., *MEMB12*. The *MEMB12* knockout mutants demonstrated augmented immunity to both avirulent and virulent strains of *P. syringae*. The plant secretory machinery is designated as vital machinery in plant–microbe interactions due to the secretion of antimicrobial proteins and several other biomolecules. During loss-of-function studies involving MEMB12, main antimicrobial PR protein i.e., PR1 was highly secreted. This finding proposes improved resistance due to mutant *memb12* as a consequence of PR proteins secretion and accumulation. As expected, miR393* overexpression also displayed increased disease resistance to *Pst avrRpt2* and high secretion and accumulation PR1 protein [151]. Despite considerable work regarding identification of miRNAs in plant defense, there are still a large number of sRNAs that pose confusion and are still unknown but, expectedly, play a key role in plant defense against fungal infections (Table 3).

siRNAs, like miRNAs, enhance gene expression reorganization in plant defense responses. These sRNAs are induced against pathogens and are involved in triggering resistance to diseases. siRNAs that are involved in plant immunity include nat-siRNA, nat-siRNAATGB2, and the bacteria-induced long siRNAs, mainly AtlsiRNA-1 [50,53,56,62]. Five lsiRNAs are induced against *Pst* (*avrRpt2*) infection [56]. *AtlsiRNA*-1 is the most functionally characterized lsiRNA. This is generated from the overlapping region of the *SRRLK* (putative leucine-rich repeat receptor-like protein kinase) AtlsiRNA-1 is complementary to the 3′ UTR of the antisense gene *AtRAP.* In mutant *AtRAP*, plants reduced virulent and avirulent *Pst* growth was observed. This suggests that the role of a negative regulator in plant resistance responses. Based on such findings, it can be concluded that AtlsiRNA-1 may promote resistance against *Pst avrRpt2* infection due to the particular regulation of its target. However, the role of siRNAs in plant immunity during bacterial infections, in connection with the knowledge generated by other sRNAs, may highlight other sRNA-regulating bacteria stress responses.

## 6. Plant Small RNAs against Nematode Infections

Silencing parasitism or housekeeping-involved genes in nematode-mediated plant diseases may alter the expression of sRNA and modulate resistance to the nematode attack [49,211,212,213]. Sindhu et al. [214] studied four parasitism-involved genes for *Heterodera schachtii*. Studying different RNAi lines revealed that incomplete resistance was achieved, but the number of nematode females decreased by 23–64%. Ibrahim et al. [215] achieved reduction in the *Meloidogyne incognita* gall formation in soybean roots by suppressing the tyrosine phosphatase (TP) genes.

miRNAs also take part in plant–nematode interactions. Against the nematode, *H. schachtii*, different miRNAs were down-regulated, e.g., miR161, miR164, miR167a, miR172c, miR396c, miR396a,b, and miR398a [216,217]. Comparative miRNA profiling in *G. max* exhibited 101 miRNAs responsive to the infection of the most destructive soybean cyst nematode (SCN). Furthermore, 20 differentially expressed miRNAs were observed in SCN-resistant and susceptible *G. max* varieties [218]. Besides, nematode-induced miRNAs and sRNAs seemingly participate in the establishment and parasitism of feeding sites, respectively [216]. Overexpression of nematode-induced miRNAs, and/or silencing of their corresponding targets, may offer significant information about plant–nematode parasitism and lead to the development of crop plants with nematode resistance.

## 7. Conclusions and Future Prospects

The implication of sRNAs in controlling plant immunity and coping with virulent pathogens urges researchers to take advantage of RNA silencing machinery in terms of amplified plant immunity against diseases. Although substantial efforts are being performed globally to recognize the protection purpose of plant sRNAs, the exact anti-pathogenic defense part of their function is unclear. Similarly, siRNAs are not receiving the attention that is due for their function in anti-microbial immunity. It is also known that pathogens have improved their virulence by developing sRNAs. Hence, focusing virulence development and unraveling pathogen sRNAs with susceptible plant targets can assist in decoding plant–pathogen interactions. Likewise, investigation into missing links about infection-mediated sRNAs production and their fast removal can be very beneficial. We expect that further research upon sRNA-mediated plant defense against different diseases, by achieving disease resistance, will help us enhance global food security.

## Figures and Tables

**Table 1:** The defensive role of various sRNAs against virus and viroid infections.

sRNAs	Defensive Role in Plant Species	Viruses/Viroids	Target Gene	References
miR1885	*Brassica napus*	TuMV	*TIR–NBS–LRR*	[136]
miR482	*Solanum lycopersicum*	TCV, CMV, TRV	*NBS-LRR*	[137]
miR168	*Oryzae sativa*, *Arabidopsis*, *N. benthamiana*	RSV, RDV	*AGO1*	[138,139,140]
miR6019/miR6020	*N. tabacum*	TMV	*TIR-NBS-LRR*	[131]
miR162	*Arabidopsis*	CMV	*DCL1*	[141,142]
miR158	*B. napus*, *B. rapa*	TuMV	*PPR gene*	[143]
miR1885	*B. napus*, *B. rapa*	TuMV	*TIR-NBS-LRR gene*	[143]
amiR171	*N. tabacum*	CaMV	*2b*	[144]
amiR-AV1-1	*Tomato*	ToLCNDV	*AV1 and AN2*	[145]
amiR159	*Arabidopsis*	TYMV	*P69*	[146]
amiR159	*Arabidopsis*	TuMV	*HC-Pro*	[146]
miR159a	*N. benthamiana*	PPV	*P1/ HC-Pro*	[147]
miR167b	*N. benthamiana*	PPV	*P1/ HC-Pro*	[147]
miR171a	*N. benthamiana*	PPV	*P1/ HC-Pro*	[147]
Pre-miR171a	*Arabidopsis*	CMV	*3′-UTR*	[148]
Pre-miR159	*Arabidopsis*	TuMV	*P69*	[149]
miR159a	*N. tubacum*	PVY	*HC-Pro*	[150]
miR167b	*N. tubacum*	PVX	*TGBp1/p25*	[150]
miR171a	*N. tubacum*	PVX	*TGBp1/p25*	[150]
Pre-miR159a	*S. lycopersicum*	CMV	*2a, 2b*	[151]
Pre-miR159a	*N. benthamiana*	WSMoV	*L replicase gene (Conserved motifs)*	[152]
miR395	*Triticum*	WSMV	*Conserved region*	[153]
pre-miR319a	*Vitisvinifera*	GFLV	*Coat protein (CP)*	[154]
pre-miR169a	*N. benthamiana*	CLCuBuV	*V2 gene*	[155]
pre-miR319a	*S. lycopersicum*	ToLCV	*AV1, AV2*	[145]
pre-miR168a	*S. lycopersicum*	ToLCV	*AV1, AV2*	[145]
pre-miR319a	*N. benthamiana*	PVY	*CI, NIa, NIb, CP*	[156]
pre-miR159a	*Zea mays*	RBSDV	*Conserved region*	[157]
pre-miR171	*N. benthamiana*	WDV	*Conserved region*	[158]
pre-miR528	*O. sativa*	RSV	*Middle segment, 3 0 end*	[159]
pre-miR528	*O. sativa*	RBSDV	*Middle segment, 3 0 end*	[159]
pre-miR159a	*N. benthamiana*	CBSV	*P1, P3, CI, Nib and CP*	[160]
pre-miR159a	*N. benthamiana*	UCBSV	*P1, P3, CI, Nib and CP*	[160]
pre-miR159a	*N. benthamiana*	TSWV	*N, NSs*	[161]
Six amiRNAs	*N. benthamiana*	PSTVd	*Structural domains*	[162]

TuMV: *Turnip mosaic virus*; TCV: *Turnip crinkle virus*; CMV: *Cucumber mosaic virus*; TRV: *Tobacco rattle virus*; RSV: *Rice stripe virus*; RDV: *Rice dwarf virus*; TMV: *Tobacco mosaic virus*; CaMV: *Cauliflower mosaic virus*; ToLCNDV: *Tomato leaf curl new Dehli virus*; TYMV: *Turnip yellow mosaic virus*; PPV: *Plum pox virus;* PVX: *Potato virus X*; PYV: *Potato virus Y*; WSMoV: *Watermelon silver mottle virus*; WSMV: *Wheat streak mosaic virus*; GFLV: *Grapevine fanleaf virus*; CLCuBuV: *Cotton leaf curl Borewala virus*; ToLCV: *Tomato leaf curl virus*; RBSDV: *Rice black streaked dwarf virus*; WDV: *Wheat dwarf virus*; CBSV: *Cassave brown streak virus*; UCBSV: *Uganda cassava brown streak virus*; TSWV: *Tomato spotted wilt virus*; PSTVd: *Potato spindle tuber viroid.*

**Table 2:** The defensive role of various sRNAs against fungal infections.

Small RNA	Defensive Role in Plant Specie	Fungus	Target Gene	References
tae-miR408	Wheat	*Puccinia striiformis* f. sp. *tritici*	*TaCLP1, a type of plantacyanin protein*	[187]
miR396a-5p	*Solanaceae*	*P. infestans*	*GRF*	[188]
miR5300	*S. lycopersicum*	*F. oxysporum*	*Solyc05g008650, tm-2*	[138]
miR396	*Arabidopsis*	*Plectosphaerella cucumerina*	*GRF*	[189]
miR396	*Arabidopsis*	*Botrytis cinerea*	*GRF*	[189]
miR396	*Arabidopsis*	*Fusarium oxysporum* f. sp. *Conglutinans*	*GRF*	[189]
miR396	*Arabidopsis*	*Colletotrichum higginsianum*	*GRF*	[189]
miR160	*P. trichocarpa*	*Botryosphaeria dothidea*	*Auxin response factor, Aux/IAA*	[165,185]
miR160	*Pinustaeda*	*Cronartium quercuum* f. sp. *fusiforme*	*Auxin response factor, Aux/IAA*	[190]
miR482	Cotton	*V. dahlia*	*Disease resistance protein*	[163,164,181]
miR1447	*P. beijingensis*	*Dothiorella gregaria*	*Disease resistance protein*	[163,164]
miR1448	Cotton	*V. dahlia*	*Disease resistance protein*	[163,164]
miR1448	*P. beijingensis*	*D. gregaria*	*Glutathione*	[165]
miR1448	*P. trichocarpa*	*B. dothidea*	*S-conjugate, ABC transporter, ATP-binding cassette transport protein*	[181]
miR1450	*P. trichocarpa*	*B. dothidea*	*Leucine-rich repeat*	[165]
miR2118	Cotton	*V. dahlia*	*TIR-NBS-LRR*	[163]
Pbe-SR3	*P. beijingensis*	*D. gregaria*	*Leucine Rich Repeat*	[164]
Pbe-SR23	*P. beijingensis*	*D. gregaria*	*Leucine Rich Repeat, NB-ARC domain*	[164]
Pbe-SR25	*P. beijingensis*	*D. gregaria*	*FtsH Extracellular*	[164]

**Table 3:** Unknown sRNAs that are involved in the defensive role against fungal infections.

Small RNA	Defensive Role in Plant Specie	Fungus	Target Gene	References
Unknown	*S. lycopersicum*	*F. oxysporum*	*Solyc08g075630, Solyc08 g076000*	[138]
Unknown	*S. lycopersicum*	*F. oxysporum*	*Solyc05g008650, tm-2*	[138]
Unknown	Wheat	*P. striiformis* f. sp. *Tritici*	*TaCLP1, a type of plantacyanin protein*	[187]
Unknown	*Gossypium raimondii*	*V. dahlia*	*NBS-LRR*	[137]
Unknown	*Morchella esculenta*	*Colletotrichum gloeosporioides*	*ARF10*	[191]
Unknown	*O. sativa*	*Magnaporthe oryzae*	*ARF16 and aB3 DNA-binding domain-containing protein*	[12]
Unknown	*M. esculenta*	*C. gloeosporioides*	*TIR1*	[191]
Unknown	*Hordeum vulgare L.*	*Blumeria graminis* f. sp. *Hordei*	*SOD1*	[192]
Unknown	*O. sativa*	*M. oryzae*	*SOD2*	[12]
Unknown	*M. esculenta*	*C. gloeosporioides*	*ARF10*	[191]

**Table 4:** The defensive role of various sRNAs against bacterial infections.

Small RNA	Defensive Role in Plant Specie	Bacteria	Target Gene	References
miR159	*Arabidopsis*	*P. syringae*	*MYB33*	[197]
miR159	*Arabidopsis*	*P. syringae*	*MYB65*	[197]
miR159	*Arabidopsis*	*P. syringae*	*MYC101*	[197]
miR160	*Arabidopsis*	*P. syringae*	*ARF10*	[198]
miR160	*Arabidopsis*	*P. syringae*	*ARF16*	[198]
miR160	*Arabidopsis*	*P. syringae*	*ARF17*	[198]
miR167	*Arabidopsis*	*P. syringae*	*ARF8*	[199]
miR167	*Arabidopsis*	*P. syringae*	*ARF6*	[197]
miR393	*Arabidopsis*	*P. syringae*	*TIR1*	[194]
miR393	*Arabidopsis*	*P. syringae*	*AFB2*	[199]
miR393	*Arabidopsis*	*P. syringae*	*AFB3*	[199]
miR393b	*Arabidopsis*	*P. syringae*	*MEMB12*	[58]
miR393b	*N. benthamiana*	*P. syringae*	*MEMB12*	[58]
miR399	*Citrus*	*Candidatus liberibacter. asiaticus*	*PHO2*	[200]
miR408	*Arabidopsis*	*P. syringae*	*Copper protein plantacyanin and copper ion binding protein genes*	[197]
miR825	*Arabidopsis*	*P. syringae*	*Remorin, zinc finger homebox family, frataxin-related*	[199]
nat-siRNA -ATGB2	*Arabidopsis*	*P. syringae*	*PPRL*	[62]
AtlsiRNA-1	*Arabidopsis*	*P. syringae*	*AtRAP*	[56]
miR390	*Arabidopsis*	*P. syringae*	*TAS3*	[197]
miR398	*Arabidopsis*	*P. syringae*	*COX5b.1*	[182]
miR398	*Arabidopsis*	*P. syringae*	*CSD1*	[198]
miR398	*Arabidopsis*	*P. syringae*	*CSD2*	[198]
miR408	*Arabidopsis*	*P. syringae*	*Copper protein plantacyanin, laccase copper protein and copper ion binding protein genes (predicted targets)*	[197]
miR773	*Arabidopsis*	*P. syringae*	*MET2*	[198]
miR472	*Arabidopsis*	*P. syringae*	*CC-NBS-LRR*	[201]
